# Single-center experience with catheter-directed thrombolysis and balloon angioplasty for acute upper-extremity deep vein thrombosis: a case series study

**DOI:** 10.1186/s12872-023-03389-3

**Published:** 2023-07-17

**Authors:** Yaser Jenab, Saeed Tofighi, Aryan Ayati, Ali Rezvanimehr, Najme-sadat Moosavi, Houman Jalaie, Mohammad Esmaeil Barbati

**Affiliations:** 1 Cardiovascular Diseases Research Institute, Tehran Heart Center, Tehran University of Medical Sciences, Tehran, Iran; 2 Faculty of Medicine, Tehran Medical Sciences Branch, Islamic Azad University, Tehran, Iran; 3 Department of Vascular and Endovascular Surgery, University Hospital Aachen, Aachen, Germany

**Keywords:** Upper Extremity Deep vein thrombosis, Balloon angioplasty, Thrombolytic therapy

## Abstract

**Background:**

Effective treatment of upper extremity deep vein thrombosis (UEDVT) is crucial to prevent further complications. Various treatments, including percutaneous mechanical thrombectomy (PMT), catheter-directed thrombolysis (CDT), decompression surgery, and venoplasty are suggested for UEDVT. However, no prospective study has yet favored any of these treatments. This study presents a review of our experience with CDT followed by balloon venoplasty in patients with acute primary UEDVT.

**Methods:**

We enrolled all patients diagnosed with acute UEDVT from January 2020 to June 2021. Subjects with UEDVT due to secondary causes like malignancies, indwelling catheters, or leads were excluded. CDT was performed through brachial vein access, using a perfusion catheter, and rt-PA administration. Balloon venoplasty was performed if the treated segment had remaining stenosis after CDT. Patients were followed up at the vein clinic for any signs and symptoms in the upper extremity and lifestyle changes. Follow-up ultrasonography was done 12 months after discharge.

**Results:**

Twelve patients with a mean age of 41.08 ± 14.0 years were included in the study. The mean duration of CDT was 25.00 ± 10.56 h. After CDT, all patients had remaining occlusions, with seven having more than 50% remaining stenosis. However, after balloon venoplasty, no patient had significant (more than 50%) stenosis. There was no serious complication after both procedures. Patients were followed up for a mean duration of twelve months after their admission, with a mean time of maintenance anticoagulation was 10.73 ± 5.77 months. Only one patient had recurrent symptoms in his target limb which required a decompression surgery, while the rest were free of symptoms in their treated extremity. No subject developed pulmonary emboli (PE) during admission or the follow-up period. There was no evidence of hospital readmission for any reason. Upper extremity color-doppler sonography of the patients at twelve months after their procedure showed normal venous flow without any significant stenosis in 8 (66.7%), and partially normal flow with patent target vein in 4 (33.3%) patients.

**Conclusions:**

CDT followed by balloon venoplasty may be an effective treatment for selected patients with acute primary UEDVT, providing desirable long-term results and potentially avoiding the need for decompression surgery in the short or long term.

**Supplementary Information:**

The online version contains supplementary material available at 10.1186/s12872-023-03389-3.

## Background

Upper extremity deep vein thrombosis (UEDVT) is a common problem, occurring in 1.6% of hospitalized patients [1], and accounts for 5–6% of all deep vein thromboses (DVTs) [2, 3]. UEDVT typically involves the subclavian, axillary, and brachial veins [4].

Primary UEDVT is a rare condition with 1–2 per 100,000 patients occurrence each year [5]. The most common cause of primary UEDVT is Paget-Schroetter syndrome (PSS), with 10–28% of cases associated with a history of excessive upper limb effort [6]. Secondary UEDVT is commonly caused by indwelling central venous catheters, cancer, or pacemaker catheters [7].

UEDVT can lead to various complications such as pulmonary emboli (PE) in up to one-third of cases [4, 8], with PE occurring more often in secondary forms [9]. Post-thrombotic syndrome (PTS) occurs in 19.4% of UEDVT patients, which can cause persistent limb swelling, pain, and heaviness, as well as recurrent DVT with a prevalence of 6-7.5% [10].

Timely and adequate treatment of UEDVT is the key to preventing further complications such as recurrent DVT, PTS, and PE [2, 11]. Although there is no randomized clinical trial (RCT) on thrombolysis effectiveness in UEDVT management, thrombolysis is considered a potential adjunct treatment in selected patients with severe symptoms, subclavian-axillary vein thrombosis, good functional status, and low risk of bleeding [12]. Other interventional treatments, such as percutaneous mechanical thrombectomy (PMT), catheter-directed thrombolysis (CDT), decompression surgery (i.e., first rib resection), and venoplasty are suggested in the UEDVT treatment [11]. But no prospective RCT has yet favored any of these treatments over the others [13–16]. It has been shown that after thrombolysis, a significant number of patients have venous stenosis caused by scarring, and many will have extrinsic compression at the costoclavicular junction. These findings support thrombolysis and decompression therapy to maintain lumen patency and prevent recurrence [16].

This study presents a retrospective review of our experience with CDT followed by balloon angioplasty in patients with acute UEDVT.

## Methods

### Study design and population

This single-center cross-sectional case series was conducted at Tehran Heart Center Hospital, Tehran, Iran, from 2020 to 2021. All the patients were aware of the study process and informed consent was needed to enter. The study protocol was approved by the ethics committee of Tehran University of Medical Sciences. Twelve patients with acute UEDVT were prospectively included in the study, with inclusion criteria of the first episode of UEDVT and symptom duration (assumed to be the best estimate of thrombus age) < 14 days. Exclusion criteria were age < 18 or > 80 years old, contraindication to fibrinolytic agents, malignancy or other concomitant chronic or potentially life-threatening diseases, uncontrolled hypertension, recent surgery, and indwelling central venous catheters or implantable cardiac defibrillator (ICD)/Pacemaker leads. The initial diagnosis was made based on the clinical presentation together with a sonography examination and confirmed via invasive venography.

### Intervention and follow-up

**Fig. 1:**
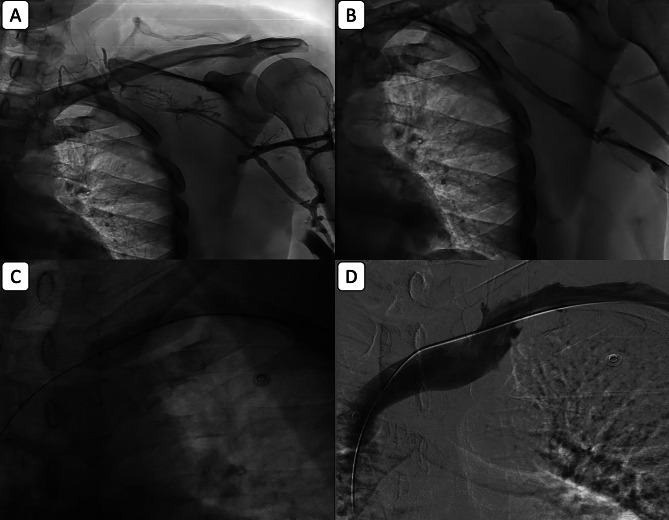
Catheter-directed thrombolysis (CDT) and balloon venoplasty in a 56-year-old male with left upper extremity pain. **(A)** Venography via the left brachial vein shows complete obstruction. **(B)** Venography after 30 h of CDT shows partial resolution of stenosis with a faint flow. **(C)** Balloon venoplasty is performed. **(D)**Final venography demonstrates complete improvement of obstruction and normal venous flow. Symptoms completely resolved post-procedure, and Doppler sonography at one-year follow-up showed no residual stenosis

All patients received unfractionated heparin (UFH) on the day of diagnosis according to local routines based on international guidelines. Venographies were performed from the ipsilateral brachial vein access (Fig. 1, A). CDT was performed with a perfusion catheter (McNamara - Medtronic), and continuous infusion of rt-PA (Alteplase) at a rate of 1 mg/h for 24–48 h until the symptom improvement (Fig. 1, B). Activated partial thromboplastin time (APTT) and fibrinogen level were measured every 12 h to adjust the UFH and Alteplase doses. After the termination of CDT, balloon venoplasty was performed if the treated segment had severe stenosis > 50%. Armada PTA Catheter balloon (Abbott Medical) was used to perform the balloon angioplasty (Fig. 1, C). Final venography after balloon venography was used for the assessment of residual stenosis and procedural success (Fig. 1, D).

Oral anticoagulant agents (Warfarin, Rivaroxaban, or Apixaban according to patient profile and preferences) were prescribed for at least 6 months, and the international normalized ratio (INR) was maintained between 2.0 and 3.0 for Warfarin. For direct thrombin inhibitors, a full-loading dose was administered (with 15 mg BD for Rivaroxaban, or 10 mg, BD for Apixaban). Maintenance dosage was subsequently ordered for the patients after the initial treatment period (20 mg, Daily Rivaroxaban, or 5 mg BD Apixaban). Patient demographics, DVT characteristics, procedural details, and complications were all collected and reviewed for each patient.

### Definitions

Technical success was defined as the absence of significant stenosis after CDT followed by balloon venoplasty, with restored patent venous blood flow. Clinical success was defined as a decrease in pain and/or swelling of the affected extremity (partial or complete) within the index hospitalization. The degree of thrombus removal was graded by calculating the percentage reduction in the patient’s total thrombus score and classified as complete clearance (100%), most clearance (50–99%), and partial clearance (< 50%).

Hemorrhage was recorded as a complication and divided into mild hemorrhage including access point hemorrhage or visible hematoma, and severe hemorrhage including intracranial hemorrhage or gastrointestinal hemorrhage.

### Follow-up

Clinical follow-up was assessed by ultrasound on the 1 and 12 months after the hospital discharge. Patients were visited at the vein clinic and checked for any signs and symptoms in the treated upper extremity, as well as any alteration in routine lifestyle.

### Statistical analysis

Numerical values are presented as mean (standard deviation), and categorical variables are summarized as frequencies (percentages). Statistical analysis was performed using the SPSS software (version 22.0; SPSS, Chicago, IL, USA).

## Results

Table S1 summarizes the baseline characteristics, procedural details, and one-year follow-up data for each patient. Twelve patients were included in this study, of whom eleven cases were male (91.7%), and the mean age of the total population was 41.08 ± 14.0 years. Table 1 provides a comprehensive summary of the procedural and follow-up outcomes for all patients.

**Table 1:** Baseline and Procedural Characteristics of All Patients

Characteristics		N = 12
***Baseline Features***		
***Age***		41.08 ± 14.0
***Gender***	*Male*	11 (91.7%)
	*Female*	1 (8.3%)
***Body temperature ©***		36.36 ± 0.56
***Heart rate (beat/min)***		89.92 ± 35.12
***Systolic blood pressure (mmHg)***		133.67 ± 29.12
***Delay from symptoms to admission (days)***		3.5 ± 3.1
***Risk Factors***	*None*	3 (25%)
	*COV*	3 (25%)
	*RMA*	5 (41.7%)
	*CS*	1 (8.3%)
***Symptoms***	*Sw.*	2 (16.7%)
	*Pn.*	2 (16.7%)
	*Sw. & Pn.*	8 (66.7%)
***Side of thrombosis***	*Right*	6 (50%)
	*Left*	6 (50%)
***CDT***		
***Venous access***	*Br.*	10 (83.3%)
	*Ce.*	1 (8.3%)
	*Br. & Fe.*	1 (8.3%)
***Mean CDT time (hours)***		25.00 ± 10.56
***Mean Alteplase dose (mg)***		23.08 ± 6.05
***CDT Results*** ***(Thrombus emoval)***	*Complete Clearance*	0 (0%)
	*Most Clearance*	5 (41.6%)
	*Partial Clearance*	7 (58.3%)
***Balloon Venoplasty***		
***Ballooning Results*** ***(Thrombus removal)***	*Complete Clearance*	12 (100%)
	*Most Clearance*	0 (0%)
	*Partial Clearance*	0 (0%)
***Anticoagulation on discharge***	*VKA*	3 (25%)
	*Riv.*	8 (66.7%)
	*Api.*	1 (8.3%)
***Complications***	*No complication*	10 (83.3%)
	*Drop in Hg (2 g)*	2 (16.7%)
	*Hematoma*	0
	*Transfusion*	0
	*Major bleeding*	0
***Follow-up Results***		
***Duration of Anticoagulation (mean, month)***		10.73 (3–21)
***Recurrent Symptoms***		1 (8.3%)
***Intermittent swelling with arm use***		2 (16.7%)
***Decompression surgery***		1 (8.3%)
***Pain relief***	*Complete*	4 (33.3%)
	*Partial*	6 (50%)
	*None*	2 (16.7%)
***Employment***	*Full*	9 (75%)
	*Limited*	3 (25%)
	*No return*	0 (0%)
***Follow-up sonography***	*Normal*	8 (66.7%)
	*Partially normal*	4 (33.3%)

Pn, Pain; Sw: Swelling; RMA, Repetitive muscular activity; COV, Covid infection/vaccination; CS, Cigarette smoker; Br, Brachial access; Fe, Femoral access; Ce, Cephalic access; VKA, Vitamin K antagonist; Riv, Rivaroxaban; Api, Apixaban

The mean delay time from symptoms onset to first medical contact was 3.5 (1-10) days. Most of the patients complained of limb swelling and pain. The number of affected upper limbs was equal between the left and right sides, each seen in six patients. The Axillary and Subclavian veins of the affected limb were involved in all of the patients. Regarding venous access for the CDT procedure, the brachial vein was used for most patients (11 out of 12), while the cephalic vein was utilized for one patient. The mean duration of CDT among all was 25.00 ± 10.56 h. Alteplase (r-tPA) was used as the thrombolytic agent for CDT in the whole population, with a mean dose of 23.08 ± 6.05 mg.

The findings of this study indicate that CDT led to unfavorable outcomes concerning residual stenosis before the balloon venoplasty, with only partial thrombus removal in 7 of the 12 patients. However, following balloon venoplasty, all patients exhibited significant stenosis improvements and complete thrombus removals. At hospital discharge, the majority of patients (8 out of 12) were prescribed Rivaroxaban, with the remainder receiving either Warfarin (3 patients) or Apixaban (1 patient) as daily maintenance anticoagulant agents. No serious complications, including mild or severe hemorrhages, were observed following either procedure. During the 12-month follow-up period, the mean maintenance anticoagulation duration was 10.73 ± 5.77 months. Only one patient had recurrent symptoms in his upper limb, while the rest remained symptom-free in their treated extremity. No subject developed PE and no hospital readmission was reported. All but three of the patients were able to return to a normal routine lifestyle without limitations or restrictions after their treatment. Technical success was observed in all patients, while clinical success was achieved in 11 out of 12 patients. In one case (Case No. 8), the patient underwent decompression surgery after CDT and balloon venoplasty due to the persistence of symptoms. Despite the surgery, the patient’s condition did not improve, and a repeated balloon venoplasty was performed, resulting in symptom resolution. The remaining 11 patients did not require surgery.

Upper extremity color-doppler sonography of the patients at 12-month follow-up showed normal venous flow without any significant stenosis in 8 (66.7%) and partially normal flow with patent target vein in 4 (33.3%) patients.

## Discussion

In this study, we investigated the efficacy of CDT followed by balloon venoplasty in twelve patients with acute UEDVT. The patients were followed up for approximately one year to evaluate target vein patency. The results revealed a high rate of target vein patency, with almost all cases demonstrating normal or partially normal venous flow. Except for one patient, decompression surgery was not necessary for any other individuals, as they had no apparent symptoms or limitations in their routine lifestyle after the procedure. The procedures were performed safely and without any major complications. These findings emphasize the significance of interventional techniques for the management of UEDVTs and suggest that balloon venoplasty may be a viable alternative to decompression surgery for such patients.

UEDVT is a rare condition that includes 5 to 10% of all DVT cases. However, with increased rates of central venous catheter insertion, its prevalence is growing recently. UEDVT mostly involves the axillary and subclavian veins, but it could happen in more distal (e.g., brachial) or proximal (e.g., jugular) veins. There are two types of UEDVT based on the underlying cause: primary UEDVT (known as Paget-Schroetter syndrome) which typically occurs in younger patients with repetitive and strenuous movements of the dominant arm and shoulder, and secondary UEDVT, which is usually caused by indwelling central venous catheters, ICD or pacemaker leads, or cancer. Nevertheless, extrinsic or internal compression is a contributor to the majority of UEDVTs, regardless of their type.

Furthermore, recent studies are suggesting hemostatic factors, including the Von Willebrand Factor (VWF), in the development, progression, and resolution of deep vein thrombosis (DVT), including UEDVT, which can include novels aspects to consider in the management of this condition[17, 18]. Other acquired causes for thrombophilia, which we assessed in this study, can also contribute to this disorder including COVID-19 infection[19, 20] or vaccinations[21, 22], smoking[23], and repetitive muscle activity[24, 25].

The management of UEDVT depends on its type. For the secondary UEDVT, routine catheter removal is not recommended. But removal is advised in cases of catheter malfunction, infection, or when it is no longer needed. Kovacs et al. studied 74 cancer patients with acute UEDVT, who were managed with dalteparin and Warfarin (INR: 2–3), and the central catheters were not removed. At three months of follow-up, there was no evidence of venous thrombosis recurrence in any of the patients [26]. However, the treatment of primary UEDVT is more complex and consists of multiple approaches, including anticoagulation, systemic or catheter-directed thrombolysis, pharmacomechanical thrombolysis, and decompression surgery. In a study by Sabeti et al., 95 patients with acute UEDVT were treated with either systemic thrombolysis (urokinase) or anticoagulation alone. The subjects who received systemic thrombolysis had a 60% reduced risk for recurrence of subclavian-axillary thrombosis at 40 months of follow-up, compared to those treated with anticoagulation alone [27]. In another study, Vik et al. conducted a retrospective cohort study of thirty patients with acute UEDVT to examine the efficacy, complications, and long-term results after CDT. The median duration of CDT was 70 h (range: 24–264), and the median dose of rt-PA was 52 mg (range: 19–225). Thrombosis lysis of more than 50% was achieved in 29 subjects (97%) immediately after the end of CDT. In our study, none of the subjects had more than 50% patency in their target veins at this time, possibly due to the lower mean duration of CDT (25 h) and dose of rt-PA (23.08 mg) compared to other studies. At follow-up (n = 29, with a median of 21 months), 11 subjects had completely thrombosed veins (38%), while the rest 18 patients (62%) had normal (n = 10) or partially normal (n = 8) venous ultrasonography. None of the patients developed severe PTS, but six (21%) of them had mild symptoms of PTS [28]. Vazquez et al. performed a systematic review of 25 studies to compare the effects of anticoagulation alone versus decompression surgery with or without thrombolysis on the development of PTS in UEDVT patients. They concluded that patients who underwent surgery had significantly lower rates of PTS compared to those treated with the anticoagulation alone [29]. In another systematic review and meta-analysis of 25 studies, Karaolanis et al. evaluated the treatment of patients with Paget-Schroetter syndrome. Of 1511 patients included in the studies, 1177 (77.9%) received thrombolysis, 658 (43.5%) were treated with anticoagulation, and 1293 (86.5%) underwent decompression surgery. At follow-up, the overall rate of subjects with no remaining stenosis in their target veins, regardless of the treatment modality, was 51.75%, whereas 84.87% of the total population were symptom-free. A subgroup meta-analysis indicated that patients who underwent first-rib resection surgery, with or without venoplasty, had a significantly higher rate of vein patency and resolution of symptoms [30].

Limitations:

While this study provides interesting insights, several limitations are worth noting. First, the sample size of participants is relatively small, not very diverse, and no control group was included due to the nature of the study, potentially limiting the generalizability of the findings. Second, the follow-up period of twelve months may not be sufficient to evaluate the long-term outcomes of the treatment, and longer-term follow-up studies are needed. Finally, the study was conducted in a single center, which may limit the applicability of the findings to other clinical settings.

## Conclusions

The finding of the current study suggests that CDT followed by balloon venoplasty may be an effective treatment for selected patients with acute UEDVT, with desirable long-term results that could potentially eliminate the need for decompression surgery in the short or long-term follow-up. However, further studies, especially RCTs are needed to confirm these results.

## Electronic supplementary material

Below is the link to the electronic supplementary material.

Additional File 1: Baseline and Procedural Characteristics and Follow-up Results of Individual Patients

## Data Availability

The data that support the findings of this study are available on request from the corresponding author (ST). The data are not publicly available due to containing information that could compromise research participant privacy. Requests to access these datasets should be directed to ST, saeedtofighi69@gmail.com.

## Declarations

### Human experimentation compliance

The scheme and design of this study were assessed and confirmed by the scientific committee of Tehran Heart Center, Tehran University of Medical Sciences. All procedures were reviewed according to the standard guidelines and regulations and approved by the Tehran Heart Center institutional scientific board.

## List of abbreviations

UEDVT: upper extremity deep vein thrombosis
DVT: deep vein thromboses
PMT: percutaneous mechanical thrombectomy
CDT: catheter-directed thrombolysis
PSS: Paget-Schroetter syndrome
PE: pulmonary emboli
RCT: randomized clinical trial
ICD: implantable cardiac defibrillator
UFH: unfractionated heparin
