# Supplementary material for: Single-center experience with catheter-directed thrombolysis and balloon angioplasty for acute upper-extremity deep vein thrombosis: a case series study
Source: BMC Cardiovasc Disord. 2023 Jul 17;23:351. doi: 10.1186/s12872-023-03389-3 (PMC10353106; doi:10.1186/s12872-023-03389-3)
Supplement: Supplementary file 1 — Additional File 1: Baseline and Procedural Characteristics and Follow-up Results of Individual Patients [file 12872_2023_3389_MOESM1_ESM.docx]

| **Table S1. Baseline and Procedural Characteristics and Follow-up Results of Individual Patients** | | | | | | | | | | | | |
| --- | --- | --- | --- | --- | --- | --- | --- | --- | --- | --- | --- | --- |
| **Case** | **1** | **2** | **3** | **4** | **5** | **6** | **7** | **8** | **9** | **10** | **11** | **12** |
| **Baseline Characteristics** | | | | | | | | | | | | |
| **Age** | 41 | 41 | 56 | 72 | 26 | 43 | 23 | 29 | 53 | 41 | 29 | 39 |
| **Gender** | Female | Male | Male | Male | Male | Male | Male | Male | Male | Male | Male | Male |
| **Time from symptoms onset to lysis (days)** | 7 | 2 | 1 | 10 | 2 | 1 | 1 | 1 | 10 | 1 | 2 | 4 |
| **Limb side** | Right | Right | Left | Left | Left | Right | Right | Left | Left | Left | Right | Right |
| **Symptoms** | Pn, Sw | Sw | Pn, Sw | Sw | Pn | Pn, Sw | Pn, Sw | Pn, Sw | Pn, Sw | Pn | Pn, Sw | Pn, Sw |
| **Risk factors** | None | RMA | COV | RMA | RMA | None | CS | RMA | RMA | COV | COV | None |
| **Procedural Characteristics** | | | | | | | | | | | | |
| **Venous access** | Br | Br | Br | Br | Br | Br | Br | Br, Fe | Br | Br | Br | Ce |
| **Duration of lysis (hours)** | 18 | 24 | 30 | 40 | 14 | 18 | 40 | 40 | 22 | 8 | 24 | 22 |
| **Alteplase dose (mg)** | 20 | 24 | 30 | 27 | 16 | 20 | 30 | 30 | 24 | 10 | 24 | 22 |
| **Thrombus Removal after CDT (%)** | Partial  Clearance | Most Clearance | Partial Clearance | Most Clearance | Partial Clearance | Most Clearance | Partial Clearance | Most Clearance | Partial Clearance | Partial Clearance | Partial Clearance | Most Clearance |
| **Thrombus Removal after Ballooning (%)** | Complete Clearance | Complete Clearance | Complete Clearance | Complete Clearance | Complete Clearance | Complete Clearance | Complete Clearance | Complete Clearance | Complete Clearance | Complete Clearance | Complete Clearance | Complete Clearance |
| **Anticoagulation on discharge** | VKA | Riv | Api | Riv | Riv | VKA | Riv | Riv | Riv | Riv | Riv | VKA |
| **Complication** | No | No | No | No | No | No | No | No | No | No | Drop in Hg | Drop in Hg |
| **Follow up** | | | | | | | | | | | | |
| **Recurrent symptoms** | No | No | No | No | No | No | No | No | No | No | No | Yes |
| **Intermittent swelling** | No | No | No | No | No | No | No | Yes | No | No | No | Yes |
| **Pain relief** | No | Partial | Complete | Partial | Partial | No | Complete | Partial | Partial | Complete | Complete | Partial |
| **Return to work** | Full | Full | Full | Limited | Limited | Full | Full | Full | Limited | Full | Full | Full |
| **Decompression surgery** | No | No | No | No | No | No | No | Yes | No | No | No | No |
| **Follow-up sonography** | Normal | Normal | Normal | Partially normal | Normal | Normal | Normal | Partially normal | Partially normal | Normal | Normal | Partially normal |

Pn, Pain; Sw: Swelling; RMA, Repetitive muscular activity**;** COV, Covid infection/vaccination; CS, Cigarette smoker**;** Br, Brachial access; Fe, Femoral access; Ce, Cephalic access; VKA, Vitamin K antagonist; Riv, Rivaroxaban; Api, Apixaban
